# Piperidinium 4-hydr­oxy-3-methoxy­carbon­yl-1,2-benzothia­zin-2-ide 1,1-dioxide

**DOI:** 10.1107/S1600536809036204

**Published:** 2009-09-12

**Authors:** Muhammad Nadeem Arshad, Muhammad Zia-ur-Rehman, Naveed Ahmed, Islam Ullah Khan

**Affiliations:** a Department of Chemistry, Government College University, Lahore-54000, Pakistan; b Applied Chemistry Research Centre, PCSIR Laboratories Complex, Ferozpure Road, Lahore 54600, Pakistan; c FC College (a Chartered University), Ferozpur Road, Lahore 54600, Pakistan

## Abstract

In the anion of the title compound, C_5_H_12_N^+^·C_10_H_8_NO_5_S^−^, the thia­zine ring adopts a distorted half-chair conformation and the enolic H atom is involved in an intra­molecular O—H⋯O hydrogen bond, forming a six-membered ring. The anions and cations are connected *via* N—H⋯N and N—H⋯O inter­actions.

## Related literature

For the synthesis of related mol­ecules, see: Zia-ur-Rehman *et al.* (2005 ▶, 2006 ▶); Braun (1923 ▶). For the biological activity of 1,2-benzothia­zine1,1-dioxides, see: Bihovsky *et al.* (2004 ▶); Turck *et al.* (1996 ▶); Zia-ur-Rehman *et al.* (2009 ▶). For related structures, see: Golič & Leban (1987 ▶).
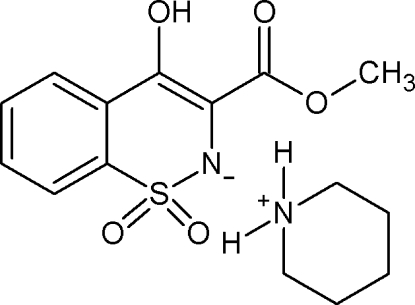


## Experimental

### 

#### Crystal data

C_5_H_12_N^+^·C_10_H_8_NO_5_S^−^
*M* *_r_* = 340.39
Monoclinic, 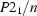

*a* = 12.0423 (5) Å
*b* = 9.1791 (3) Å
*c* = 14.5193 (5) Å
β = 90.556 (2)°
*V* = 1604.85 (10) Å^3^
*Z* = 4
Mo *K*α radiation
μ = 0.23 mm^−1^
*T* = 296 K
0.39 × 0.33 × 0.29 mm

#### Data collection

Bruker KAPPA APEXII CCD diffractometer
Absorption correction: multi-scan (*SADABS*; Bruker 2007 ▶) *T*_min_ = 0.921, *T*_max_ = 0.940
16000 measured reflections
3690 independent reflections
2896 reflections with *I* > 2σ(*I*)
*R*_int_ = 0.025

#### Refinement

*R*[*F* ^2^ > 2σ(*F* ^2^)] = 0.036
*wR*(*F* ^2^) = 0.103
*S* = 1.03
3690 reflections
216 parameters
H atoms treated by a mixture of independent and constrained refinement
Δρ_max_ = 0.30 e Å^−3^
Δρ_min_ = −0.34 e Å^−3^

### 

Data collection: *APEX2* (Bruker, 2007 ▶); cell refinement: *SAINT* (Bruker, 2007 ▶); data reduction: *SAINT*; program(s) used to solve structure: *SHELXS97* (Sheldrick, 2008 ▶); program(s) used to refine structure: *SHELXL97* (Sheldrick, 2008 ▶); molecular graphics: *PLATON* (Spek, 2009 ▶) and *Mercury* (Macrae *et al.*, 2006 ▶); software used to prepare material for publication: *WinGX* (Farrugia, 1999 ▶) and *PLATON*.

## Supplementary Material

Crystal structure: contains datablocks I, New_Global_Publ_Block. DOI: 10.1107/S1600536809036204/bt5055sup1.cif

Structure factors: contains datablocks I. DOI: 10.1107/S1600536809036204/bt5055Isup2.hkl

Additional supplementary materials:  crystallographic information; 3D view; checkCIF report

## Figures and Tables

**Table 1:** Hydrogen-bond geometry (Å, °)

*D*—H⋯*A*	*D*—H	H⋯*A*	*D*⋯*A*	*D*—H⋯*A*
O3—H3⋯O4	0.82	1.86	2.577 (2)	145
N1—H1*N*⋯N2^i^	0.932 (19)	1.951 (19)	2.881 (2)	175.4 (18)
N1—H2*N*⋯O2^ii^	0.87 (2)	1.91 (2)	2.7739 (18)	172.4 (18)

Symmetry codes: (i) 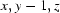; (ii) 
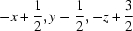
.

**Fig. 1.**
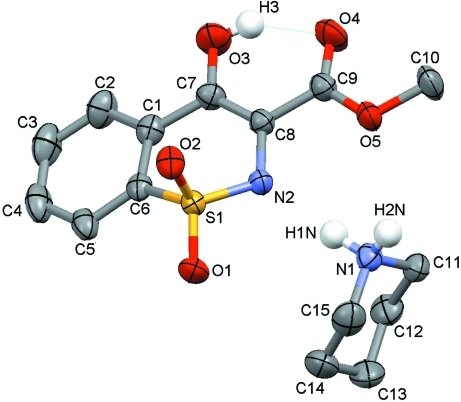
The molecular structure of the title compound, with displacement ellipsoids at the 50% probability level. Hydrogen atoms bonded to C omitted for clarity.

**Fig. 2.**
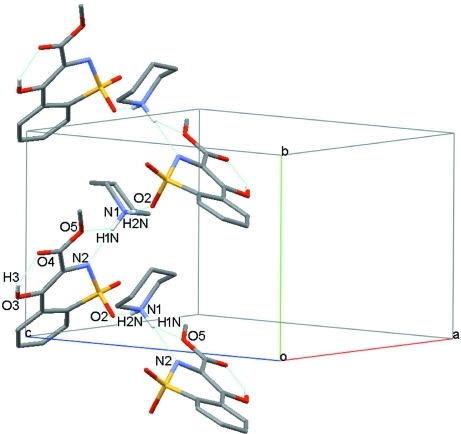
Perspective view of the three-dimensional crystal packing showing hydrogen-bonded interactions (dashed lines). H atoms not involved in hydrogen bonding have been omitted for clarity.

## supplementary crystallographic information

### Comment

Owing to their application as non-steroidal anti-inflammatory agents (Turck *et al.*, 1996), considerable attention has been given to synthetic and structural investigations of 1,2-benzothiazine1,1-dioxides and their precursor intermediates (Golič & Leban, 1987). These are known to possess a versatile range of biological activities and have been synthesized continuously since the very first synthesis (Braun, 1923). Among these, Piroxicam (Zia-ur-Rehman *et al.*, 2005), and Meloxicam (Turck *et al.*, 1996) are familiar for their analgesic action and are being used world wide as non-steroidal anti-inflammatory drugs (NSAIDs). Besides, these have also been found to be used for the treatment of rheumatoid arthritis, ankylosing spondylitis, osteoarthrosis and other inflammatory rheumatic and non- rheumatic processes, including onsets and traumatologic lesions. Some of the 3,4-dihydro-1,2-benzothiazine-3-carboxylate 1,1-dioxide α-ketomide and P(2)—P(3) peptide mimetic aldehyde compounds act as potent calpain I inhibitors (Bihovsky *et al.*, 2004) while 1,2-benzothiazin-3-yl-quinazolin-4(3*H*)-ones possess anti-bacterial properties (Zia-ur-Rehman *et al.*, 2006). As part of a research program synthesizing various bioactive benzothiazines (Zia-ur-Rehman *et al.*, 2005, 2006, 2009), we, herein report the crystal structure of the title compound (Scheme and figure 1). The asymmetric unit contains one piperidinum cation and one 4-hydroxy-3-(methoxycarbonyl)-1,2-benzothiazin-2-ide 1,1-dioxide anion. The piperidinium cation displays a typical chair conformation. The thiazine ring of the anion, involving two double bonds, exhibits a distorted half-chair conformation and the enolic hydrogen on O1 is involved in intramolecular hydrogen bonding giving rise to a six membered hydrogen bond ring (Table 1). Both the ions are linked together through N—H···N and N—H···O interactions. Adjacent asymmetric units are linked through intermolecular N—H···O hydrogen bonds, resulting in zigzag chains lying along the *b* axis (Figure 2).

### Experimental

A mixture of methyl 4-hydroxy-2*H*-1,2-benzothiazine-3-carboxylate-1,1-dioxide (2.693 g; 10.0 mmoles), piperidine (1.02 g, 12.0 mmoles) and toluene (25.0 ml) was heated to reflux for an hour. Solvent and excess piperdine were removed under vacuum and the resulting solids were dried and crystallized from ethanol. Yield: 78%.

### Refinement

All hydrogen atoms were identified in the difference map. Those bonded to O and C were fixed in ideal positions and treated as riding on their parent atoms. The following distances were used: Methyl C—H 0.98 Å. °, aromatic C—H 0.95 Å and   O—H 0.84 Å. U(H) was set to 1.2Ueq of the parent atoms or 1.5Ueq for methyl groups. The coordinates of the H atom bonded to N were refined.

### Crystal data

**Table:**

C_5_H_12_N^+^·C_10_H_8_NO_5_S^−^	*F*(000) = 720
*M**_r_* = 340.39	*D*_x_ = 1.409 Mg m^−^^3^
Monoclinic, *P*2_1_/*n*	Mo *K*α radiation, λ = 0.71073 Å
Hall symbol: -P 2yn	Cell parameters from 6881 reflections
*a* = 12.0423 (5) Å	θ = 2.6–27.4°
*b* = 9.1791 (3) Å	µ = 0.23 mm^−^^1^
*c* = 14.5193 (5) Å	*T* = 296 K
β = 90.556 (2)°	Needle, white yellow
*V* = 1604.85 (10) Å^3^	0.39 × 0.33 × 0.29 mm
*Z* = 4	

### Data collection

**Table:**

Bruker KAPPA APEXII CCD diffractometer	3690 independent reflections
Radiation source: fine-focus sealed tube	2896 reflections with *I* > 2σ(*I*)
graphite	*R*_int_ = 0.025
φ and ω scans	θ_max_ = 27.5°, θ_min_ = 2.2°
Absorption correction: multi-scan (*SADABS*; Bruker 2007)	*h* = −15→15
*T*_min_ = 0.921, *T*_max_ = 0.940	*k* = −11→11
16000 measured reflections	*l* = −18→18

### Refinement

**Table:**

Refinement on *F*^2^	Primary atom site location: structure-invariant direct methods
Least-squares matrix: full	Secondary atom site location: difference Fourier map
*R*[*F*^2^ > 2σ(*F*^2^)] = 0.036	Hydrogen site location: inferred from neighbouring sites
*wR*(*F*^2^) = 0.103	H atoms treated by a mixture of independent and constrained refinement
*S* = 1.03	*w* = 1/[σ^2^(*F*_o_^2^) + (0.0492*P*)^2^ + 0.5427*P*] where *P* = (*F*_o_^2^ + 2*F*_c_^2^)/3
3690 reflections	(Δ/σ)_max_ = 0.001
216 parameters	Δρ_max_ = 0.30 e Å^−^^3^
0 restraints	Δρ_min_ = −0.34 e Å^−^^3^

### Special details

**Table:**

Geometry. All e.s.d.'s (except the e.s.d. in the dihedral angle between two l.s. planes) are estimated using the full covariance matrix. The cell e.s.d.'s are taken into account individually in the estimation of e.s.d.'s in distances, angles and torsion angles; correlations between e.s.d.'s in cell parameters are only used when they are defined by crystal symmetry. An approximate (isotropic) treatment of cell e.s.d.'s is used for estimating e.s.d.'s involving l.s. planes.
Refinement. Refinement of *F*^2^ against ALL reflections. The weighted *R*-factor *wR* and goodness of fit *S* are based on *F*^2^, conventional *R*-factors *R* are based on *F*, with *F* set to zero for negative *F*^2^. The threshold expression of *F*^2^ > σ(*F*^2^) is used only for calculating *R*-factors(gt) *etc*. and is not relevant to the choice of reflections for refinement. *R*-factors based on *F*^2^ are statistically about twice as large as those based on *F*, and *R*- factors based on ALL data will be even larger.

### Fractional atomic coordinates and isotropic or equivalent isotropic displacement parameters (Å^2^)

**Table:**

	*x*	*y*	*z*	*U*_iso_*/*U*_eq_	
S1	0.46805 (3)	0.74091 (4)	0.76725 (3)	0.02947 (12)	
O1	0.48950 (11)	0.80132 (14)	0.85705 (8)	0.0461 (3)	
O2	0.38877 (9)	0.62124 (13)	0.76755 (8)	0.0388 (3)	
O3	0.51396 (13)	0.69087 (18)	0.47854 (8)	0.0570 (4)	
H3	0.4706	0.7429	0.4497	0.085*	
O4	0.36167 (13)	0.88585 (17)	0.45879 (8)	0.0598 (4)	
O5	0.30030 (10)	1.00194 (13)	0.58304 (8)	0.0428 (3)	
N1	0.33595 (12)	0.12173 (16)	0.77625 (10)	0.0354 (3)	
H2N	0.2658 (17)	0.113 (2)	0.7631 (13)	0.042*	
H1N	0.3719 (16)	0.041 (2)	0.7515 (13)	0.042*	
N2	0.43544 (11)	0.86485 (14)	0.69844 (9)	0.0334 (3)	
C1	0.59021 (13)	0.64809 (18)	0.62624 (12)	0.0358 (4)	
C2	0.67446 (15)	0.5623 (2)	0.58853 (15)	0.0502 (5)	
H2A	0.6768	0.5474	0.5252	0.060*	
C3	0.75373 (15)	0.4998 (2)	0.64477 (17)	0.0576 (6)	
H3A	0.8079	0.4405	0.6191	0.069*	
C4	0.75421 (15)	0.5235 (2)	0.73845 (17)	0.0536 (5)	
H4	0.8104	0.4843	0.7752	0.064*	
C5	0.67134 (14)	0.60531 (19)	0.77759 (14)	0.0425 (4)	
H5	0.6711	0.6216	0.8408	0.051*	
C6	0.58827 (12)	0.66317 (17)	0.72195 (11)	0.0322 (3)	
C7	0.50910 (14)	0.72383 (19)	0.56940 (11)	0.0359 (4)	
C8	0.43901 (13)	0.82600 (18)	0.60482 (10)	0.0319 (3)	
C9	0.36534 (14)	0.90591 (19)	0.54163 (11)	0.0366 (4)	
C10	0.22478 (18)	1.0821 (2)	0.52354 (13)	0.0553 (5)	
H10A	0.1749	1.0154	0.4934	0.083*	
H10B	0.2664	1.1342	0.4781	0.083*	
H10C	0.1829	1.1499	0.5597	0.083*	
C11	0.37737 (15)	0.25674 (18)	0.73166 (12)	0.0396 (4)	
H11A	0.3354	0.3399	0.7534	0.047*	
H11B	0.3670	0.2498	0.6655	0.047*	
C12	0.49815 (17)	0.2779 (2)	0.75380 (14)	0.0483 (5)	
H12A	0.5242	0.3667	0.7249	0.058*	
H12B	0.5404	0.1971	0.7292	0.058*	
C13	0.51701 (19)	0.2874 (2)	0.85636 (15)	0.0592 (6)	
H13A	0.4815	0.3743	0.8800	0.071*	
H13B	0.5960	0.2945	0.8694	0.071*	
C14	0.47016 (19)	0.1542 (2)	0.90404 (13)	0.0560 (5)	
H14A	0.5143	0.0697	0.8880	0.067*	
H14B	0.4758	0.1676	0.9702	0.067*	
C15	0.35025 (17)	0.1258 (2)	0.87800 (13)	0.0517 (5)	
H15A	0.3268	0.0336	0.9040	0.062*	
H15B	0.3038	0.2020	0.9033	0.062*	

### Atomic displacement parameters (Å^2^)

**Table:**

	*U*^11^	*U*^22^	*U*^33^	*U*^12^	*U*^13^	*U*^23^
S1	0.0313 (2)	0.0288 (2)	0.0283 (2)	0.00206 (15)	−0.00296 (14)	0.00160 (15)
O1	0.0628 (8)	0.0457 (7)	0.0298 (6)	0.0067 (6)	−0.0103 (5)	−0.0026 (5)
O2	0.0301 (6)	0.0365 (6)	0.0498 (7)	−0.0018 (5)	0.0027 (5)	0.0071 (5)
O3	0.0690 (10)	0.0707 (10)	0.0313 (7)	0.0207 (8)	0.0058 (6)	−0.0064 (6)
O4	0.0770 (10)	0.0738 (10)	0.0285 (6)	0.0239 (8)	−0.0077 (6)	0.0021 (6)
O5	0.0503 (7)	0.0440 (7)	0.0339 (6)	0.0158 (6)	−0.0076 (5)	0.0031 (5)
N1	0.0295 (7)	0.0335 (8)	0.0431 (8)	0.0014 (6)	−0.0020 (6)	−0.0021 (6)
N2	0.0425 (7)	0.0295 (7)	0.0280 (6)	0.0070 (6)	−0.0034 (5)	−0.0003 (5)
C1	0.0305 (8)	0.0330 (8)	0.0439 (9)	−0.0015 (7)	0.0060 (7)	0.0023 (7)
C2	0.0409 (10)	0.0493 (11)	0.0606 (12)	0.0078 (9)	0.0150 (9)	−0.0001 (10)
C3	0.0320 (9)	0.0491 (12)	0.0918 (17)	0.0096 (9)	0.0121 (10)	0.0026 (11)
C4	0.0289 (9)	0.0453 (11)	0.0863 (16)	0.0027 (8)	−0.0118 (9)	0.0105 (11)
C5	0.0342 (8)	0.0358 (9)	0.0573 (11)	−0.0029 (7)	−0.0120 (8)	0.0066 (8)
C6	0.0258 (7)	0.0268 (8)	0.0438 (9)	−0.0035 (6)	−0.0018 (6)	0.0021 (7)
C7	0.0375 (9)	0.0401 (9)	0.0301 (8)	−0.0004 (7)	0.0038 (6)	0.0003 (7)
C8	0.0350 (8)	0.0322 (8)	0.0284 (7)	−0.0001 (7)	−0.0011 (6)	0.0022 (6)
C9	0.0417 (9)	0.0363 (9)	0.0317 (8)	−0.0004 (8)	−0.0021 (7)	0.0030 (7)
C10	0.0630 (13)	0.0602 (13)	0.0426 (10)	0.0239 (10)	−0.0116 (9)	0.0095 (9)
C11	0.0444 (9)	0.0312 (9)	0.0430 (9)	−0.0001 (7)	−0.0060 (7)	0.0015 (7)
C12	0.0467 (10)	0.0394 (10)	0.0589 (12)	−0.0109 (8)	−0.0033 (9)	0.0003 (9)
C13	0.0645 (14)	0.0474 (12)	0.0653 (13)	−0.0078 (10)	−0.0238 (11)	−0.0101 (10)
C14	0.0728 (14)	0.0560 (13)	0.0389 (10)	0.0058 (11)	−0.0160 (9)	−0.0044 (9)
C15	0.0611 (12)	0.0534 (12)	0.0409 (10)	0.0075 (10)	0.0129 (9)	0.0001 (9)

### Geometric parameters (Å, °)

**Table:**

S1—O1	1.4379 (12)	C4—H4	0.9300
S1—O2	1.4554 (12)	C5—C6	1.385 (2)
S1—N2	1.5618 (13)	C5—H5	0.9300
S1—C6	1.7483 (16)	C7—C8	1.366 (2)
O3—C7	1.3553 (19)	C8—C9	1.467 (2)
O3—H3	0.8200	C10—H10A	0.9600
O4—C9	1.217 (2)	C10—H10B	0.9600
O5—C9	1.327 (2)	C10—H10C	0.9600
O5—C10	1.449 (2)	C11—C12	1.499 (3)
N1—C15	1.486 (2)	C11—H11A	0.9700
N1—C11	1.487 (2)	C11—H11B	0.9700
N1—H2N	0.87 (2)	C12—C13	1.507 (3)
N1—H1N	0.93 (2)	C12—H12A	0.9700
N2—C8	1.4063 (19)	C12—H12B	0.9700
C1—C6	1.397 (2)	C13—C14	1.516 (3)
C1—C2	1.400 (2)	C13—H13A	0.9700
C1—C7	1.450 (2)	C13—H13B	0.9700
C2—C3	1.375 (3)	C14—C15	1.512 (3)
C2—H2A	0.9300	C14—H14A	0.9700
C3—C4	1.377 (3)	C14—H14B	0.9700
C3—H3A	0.9300	C15—H15A	0.9700
C4—C5	1.376 (3)	C15—H15B	0.9700
			
O1—S1—O2	113.61 (8)	O4—C9—O5	122.16 (16)
O1—S1—N2	109.94 (7)	O4—C9—C8	123.95 (16)
O2—S1—N2	113.00 (7)	O5—C9—C8	113.88 (14)
O1—S1—C6	110.88 (8)	O5—C10—H10A	109.5
O2—S1—C6	103.81 (7)	O5—C10—H10B	109.5
N2—S1—C6	105.11 (7)	H10A—C10—H10B	109.5
C7—O3—H3	109.5	O5—C10—H10C	109.5
C9—O5—C10	115.92 (14)	H10A—C10—H10C	109.5
C15—N1—C11	112.06 (15)	H10B—C10—H10C	109.5
C15—N1—H2N	108.9 (12)	N1—C11—C12	110.10 (15)
C11—N1—H2N	108.1 (13)	N1—C11—H11A	109.6
C15—N1—H1N	110.7 (11)	C12—C11—H11A	109.6
C11—N1—H1N	109.6 (11)	N1—C11—H11B	109.6
H2N—N1—H1N	107.3 (17)	C12—C11—H11B	109.6
C8—N2—S1	115.08 (11)	H11A—C11—H11B	108.2
C6—C1—C2	117.64 (16)	C11—C12—C13	110.86 (17)
C6—C1—C7	120.06 (14)	C11—C12—H12A	109.5
C2—C1—C7	122.27 (17)	C13—C12—H12A	109.5
C3—C2—C1	120.25 (19)	C11—C12—H12B	109.5
C3—C2—H2A	119.9	C13—C12—H12B	109.5
C1—C2—H2A	119.9	H12A—C12—H12B	108.1
C2—C3—C4	121.11 (18)	C12—C13—C14	110.59 (16)
C2—C3—H3A	119.4	C12—C13—H13A	109.5
C4—C3—H3A	119.4	C14—C13—H13A	109.5
C5—C4—C3	119.85 (18)	C12—C13—H13B	109.5
C5—C4—H4	120.1	C14—C13—H13B	109.5
C3—C4—H4	120.1	H13A—C13—H13B	108.1
C4—C5—C6	119.40 (19)	C15—C14—C13	112.54 (17)
C4—C5—H5	120.3	C15—C14—H14A	109.1
C6—C5—H5	120.3	C13—C14—H14A	109.1
C5—C6—C1	121.54 (16)	C15—C14—H14B	109.1
C5—C6—S1	122.21 (14)	C13—C14—H14B	109.1
C1—C6—S1	115.89 (12)	H14A—C14—H14B	107.8
O3—C7—C8	123.54 (15)	N1—C15—C14	110.74 (15)
O3—C7—C1	114.31 (15)	N1—C15—H15A	109.5
C8—C7—C1	122.03 (15)	C14—C15—H15A	109.5
C7—C8—N2	124.26 (14)	N1—C15—H15B	109.5
C7—C8—C9	118.70 (14)	C14—C15—H15B	109.5
N2—C8—C9	116.99 (14)	H15A—C15—H15B	108.1
			
O1—S1—N2—C8	165.08 (12)	C2—C1—C7—O3	7.3 (2)
O2—S1—N2—C8	−66.84 (13)	C6—C1—C7—C8	9.0 (3)
C6—S1—N2—C8	45.69 (13)	C2—C1—C7—C8	−168.87 (17)
C6—C1—C2—C3	−2.0 (3)	O3—C7—C8—N2	−177.84 (16)
C7—C1—C2—C3	175.93 (18)	C1—C7—C8—N2	−2.0 (3)
C1—C2—C3—C4	−2.0 (3)	O3—C7—C8—C9	−0.5 (3)
C2—C3—C4—C5	3.1 (3)	C1—C7—C8—C9	175.28 (15)
C3—C4—C5—C6	−0.1 (3)	S1—N2—C8—C7	−29.7 (2)
C4—C5—C6—C1	−4.0 (3)	S1—N2—C8—C9	152.98 (12)
C4—C5—C6—S1	168.73 (14)	C10—O5—C9—O4	−0.2 (3)
C2—C1—C6—C5	5.0 (2)	C10—O5—C9—C8	−179.11 (16)
C7—C1—C6—C5	−172.97 (16)	C7—C8—C9—O4	0.8 (3)
C2—C1—C6—S1	−168.17 (13)	N2—C8—C9—O4	178.31 (17)
C7—C1—C6—S1	13.8 (2)	C7—C8—C9—O5	179.66 (15)
O1—S1—C6—C5	28.75 (16)	N2—C8—C9—O5	−2.8 (2)
O2—S1—C6—C5	−93.61 (15)	C15—N1—C11—C12	59.0 (2)
N2—S1—C6—C5	147.50 (14)	N1—C11—C12—C13	−58.6 (2)
O1—S1—C6—C1	−158.11 (12)	C11—C12—C13—C14	55.4 (2)
O2—S1—C6—C1	79.53 (13)	C12—C13—C14—C15	−52.5 (2)
N2—S1—C6—C1	−39.36 (14)	C11—N1—C15—C14	−55.5 (2)
C6—C1—C7—O3	−174.80 (15)	C13—C14—C15—N1	52.2 (2)

### Hydrogen-bond geometry (Å, °)

**Table:**

*D*—H···*A*	*D*—H	H···*A*	*D*···*A*	*D*—H···*A*
O3—H3···O4	0.82	1.86	2.577 (2)	145
N1—H1N···N2^i^	0.932 (19)	1.951 (19)	2.881 (2)	175.4 (18)
N1—H2N···O2^ii^	0.87 (2)	1.91 (2)	2.7739 (18)	172.4 (18)

Symmetry codes: (i) *x*, *y*−1, *z*; (ii) −*x*+1/2, *y*−1/2, −*z*+3/2.
